# Interactions of plumbagin with five common antibiotics against *Staphylococcus aureus in vitro*

**DOI:** 10.1371/journal.pone.0297493

**Published:** 2024-01-26

**Authors:** Songtao Bie, Qiuyue Mo, Chen Shi, Hui Yuan, Chunshuang Li, Tong Wu, Wenlong Li, Heshui Yu

**Affiliations:** 1 College of Pharmaceutical Engineering of Traditional Chinese Medicine, Tianjin University of Traditional Chinese Medicine, Tianjin, China; 2 State Key Laboratory of Component-based Chinese Medicine, Tianjin University of Traditional Chinese Medicine, Tianjin, China; 3 Haihe Laboratory of Modern Chinese Medicine, Tianjin, China; University of Jeddah, SAUDI ARABIA

## Abstract

*Staphylococcus aureus* is the main culprit, causing a variety of severe clinical infections. At the same time, clinics are also facing the severe situation of antibiotic resistance. Therefore, effective strategies to address this problem may include expanding the antimicrobial spectrum by exploring alternative sources of drugs or delaying the development of antibiotic resistance through combination therapy so that existing antibiotics can continue to be used. Plumbagin (PLU) is a phytochemical that exhibits antibacterial activity. In the present study, we investigated the in vitro antibacterial activity of PLU. We selected five antibiotics with different mechanisms and inhibitory activities against *S*. *aureus* to explore their interaction with the combination of PLU. The interaction of combinations was evaluated by the Bliss independent model and visualized through response surface analysis. PLU exhibited potent antibacterial activity, with half maximal inhibitory concentration (IC_50_) and minimum inhibitory concentration (MIC) values against *S*. *aureus* of 1.73 μg/mL and 4 μg/mL, respectively. Synergism was observed when PLU was combined with nitrofurantoin (NIT), ciprofloxacin (CPR), mecillinam (MEC), and chloramphenicol (CHL). The indifference of the trimethoprim (TMP)-PLU pairing was demonstrated across the entire dose-response matrix, but significant synergy was observed within a specific dose region. In addition, no antagonistic interactions were indicated. Overall, PLU is not only a promising antimicrobial agent but also has the potential to enhance the growth-inhibitory activity of some antibiotics against *S*. *aureus*, and the use of the interaction landscape, along with the dose-response matrix, for analyzing and quantifying combination results represents an improved approach to comprehending antibacterial combinations.

## 1. Introduction

From antiquity to the present day, humanity has been plagued by numerous infectious diseases. *Staphylococcus aureus* is a major contributor to a range of clinical infections, including bacteremia, infective endocarditis, osteoarthritis, skin infections, and implant infections [1, 2]. *S*. *aureus* has developed resistance to numerous antimicrobial agents, posing significant clinical challenges in the treatment of infectious diseases [3–5]. In this context, there remains a clinical imperative to search for new antimicrobial substances from various sources, such as medicinal plants, and to identify novel therapies with improved efficacy and diminished toxicity, such as the combined use of adjuvants and antibiotics [6–8].

Medicinal plants have been well-demonstrated as promising sources of effective antimicrobial drugs [9–11]. *Plumbago indica* Linn. is a medicinal plant that has played a significant role in ancient medicine in many countries, including China, India, and Ceylon [12]. It is well known for its traditional uses as an antimicrobial, anti-inflammatory, carminative, nerve stimulant, and rejuvenating drug [13]. What’s more, its active constituent, PLU (5-hydroxy-2-methyl-1,4-naphthoquinone), a naturally occurring naphthoquinone, has shown antibacterial effects, particularly against *S*. *aureus*. Besides its antibacterial activity, PLU also exhibits anti-inflammatory, antimalarial, antiparasitic, and anticancer properties [14, 15]. Furthermore, several compounds of 1,4-naphthoquinone, including PLU, appear to be safe for humans because these compounds do not alter the coagulation parameters in human plasma [16]. Given its proven usefulness and non-toxic nature, PLU could be a promising compound in the development of antimicrobial agents or adjuvant-antibiotic drugs.

Disappointingly, the activity of PLU, as well as other antibacterials derived from plants, is generally weaker than that of common antibiotics. However, there is evidence suggesting that PLU may enhance the effects of common antibiotics when used in combination with adjuvant compounds. Rondevaldova et al. [17] reported that PLU exhibited synergistic effects when combined with oxacillin and additive effects with tetracycline against *S*. *aureus*. Yet, after the publication of Rondevaldova, a recent report by Yap et al. [18] investigated the synergy between 1,4-naphthoquinone and selected β-lactam antibiotics against *S*. *aureus*, which showed a synergistic effect with cefotaxime, an additive effect with imipenem, and an antagonistic effect with cefuroxime.

The effect of combinations of 1,4-naphthoquinone and antibiotics is confounded by the different reference models of non-interaction and the number of distinct drugs [17, 18]. Therefore, PLU was chosen carefully as a representative compound of 1,4-naphthoquinone in order to study its interactions with five commonly used antibiotics that have different mechanisms of action.

Currently, there are many classes of reference models: The fractional inhibitory concentration index (FICI) model [19], the zero interaction potency (ZIP) model [20], the highest single agent (HSA) model, the Chou-Talalay method [21–23], the Loewe additivity model, and the Bliss independence model [24]. The major advantage of the Bliss independence model is its independence from MIC endpoints and MIC definitions. Instead of comparing concentrations, it compares the effects of drugs when administered alone or in combination [25]. This model can be a viable alternative in high-throughput screening settings when it is impractical to use large dose ranges. Therefore, the Bliss independence model was used to quantify the interaction between PLU and each of the five common antibiotics.

The aim of this study was to evaluate the combined antimicrobial efficacy of PLU and common antibiotics with different mechanisms and activities against *S*. *aureus* and to quantify the interaction between the combinations using the Bliss independent model.

## 2. Materials and methods

### 2.1. Bacteria and culture conditions

*S*. *aureus* (CMCC (B)26003) was purchased from the China Institute for Food and Drug Control. Stock cultures of the bacteria were preserved at -80˚C in Luria Bertani (LB) medium supplemented with 15% glycerol (Aladdin Reagent Ltd., Shanghai, China). For each set of assays, 10 mL of LB liquid medium was inoculated with 10 μL of the bacterial stock cultures and incubated in a 37°C incubator for 18–22 hours at 200 rpm. Before being used in assays, the *S*. *aureus* cells were diluted with LB liquid medium to an OD_625_ reading of 0.08–0.13 using a full-wavelength microplate reader (Tecan Infinite F50, Tecan Trading AG, Switzerland). The final concentration of the bacterial suspension in each well was approximately 5×10^5^ CFU/mL.

### 2.2. Plumbagin and antibiotics

PLU was purchased from Aladdin Reagent Ltd. in Shanghai, China. NIT, CPR, TMP, and CHL were obtained from Sigma-Aldrich, while MEC was sourced from Shanghai Yuanye Biological Co., Ltd. in Shanghai, China. The stock solutions of PLU and antibiotics were prepared in water (MEC) or dimethyl sulfoxide (DMSO) (NIT, CPR, TMP, CHL, and PLU) due to the varying solubility of each compound. The solutions were then passed through 0.22 μm filters and stored in the dark at -20°C. Before being used in assays, the stocks were thawed and diluted to the desired concentrations.

### 2.3. Minimum inhibitory concentration (MIC) determination

The MIC of PLU and each of the five common antibiotics for *S*. *aureus* was determined in vitro using the broth microdilution method. This method was conducted according to the guidelines set by the Clinical and Laboratory Standards Institute (CLSI, 2019) and utilized 96-well microtiter plates. Briefly, the *S*. *aureus* suspension dilutions were added to the wells of a microtiter plate. The plate contained serial 2-fold dilutions of PLU and five common antibiotics. To determine the appropriate concentration ranges for the drug dilution assay, the concentration range was selected based on published reports (Table 1). The microtiter plates were incubated at 37°C for 24 hours, and bacterial growth was measured as optical density by a full-wavelength microplate reader at 625 nm. The MICs of PLU and each of the five common antibiotics were determined as the concentration that resulted in a 90% inhibition.

**Table 1:** MIC and IC_50_ against *S*. *aureus* of PLU and five antibiotics.

Drug or Compound	Mode of action (Known target)	Concentration Range (μg/mL)	MIC (μg/mL)	IC_50_ (μg/mL)
PLU	Unknown	0.25–32	4	1.73
NIT	Interferes with oxidoreductases and prevents normal bacterial metabolism	0.5–64	16	6.10
CPR	Inhibit DNA synthesis and replication (gyrase)	0.03125–4	0.5	0.16
MEC	inhibit the synthesis of the cell wall (Penicillin Binding Protein)	0.5–64	8	2.55
TMP	Interferes with bacterial folic acid metabolism (DHFR)	2–64	8	3.50
CHL	block protein synthesis (50S ribosome subunit)	0.125–16	4	1.82

MIC: Minimum inhibitory concentration, IC_50_: half maximal inhibitory concentration, PLU: plumbagin, NIT: nitrofurantoin, CPR: ciprofloxacin, MEC: mecillinam, TMP: trimethoprim, CHL: chloramphenicol.

### 2.4. Dose-response curves and half maximal inhibitory concentration (IC_50_) values derivation

Dose-response curves were compiled in 96-well microtiter plates for PLU and each of the five common antibiotics at a concentration of 0.125×MIC–8×MIC. Each concentration was performed in triplicate wells. Appropriate serial dilutions of individual drugs were performed in LB liquid medium; the final volume per well was 100 μL. Then, 100 μL of the bacterial suspension was added to each well. Each plate also included a positive control (100 μL LB plus 100 μL bacterial suspensions), a plate blank (100 μL LB plus 100 μL serial dilutions of individual drugs), and a negative control (200 μL LB). The microtiter plates were incubated at 37°C with shaking, and bacterial growth (OD_625_) was measured after 24 hours of incubation using a full-wavelength microplate reader. The percentage of bacterial growth was then calculated according to Eq (1).


$$Bacterial\;growth\;(\%)=\frac{OD\;of\;drug\;treatment-OD\;of\;plate\;blank}{OD\;of\;positive\;control-OD\;of\;negative\;control}\times 100\%,$$ (1)

The dose responses of PLU and each of the five common antibiotics were then plotted, and the IC_50_ values were determined using GraphPad Prism 9.0 (GraphPad Software, San Diego, CA, USA).

### 2.5. *Staphylococcus aureus* two-drug dilution assay

**Fig 1:**
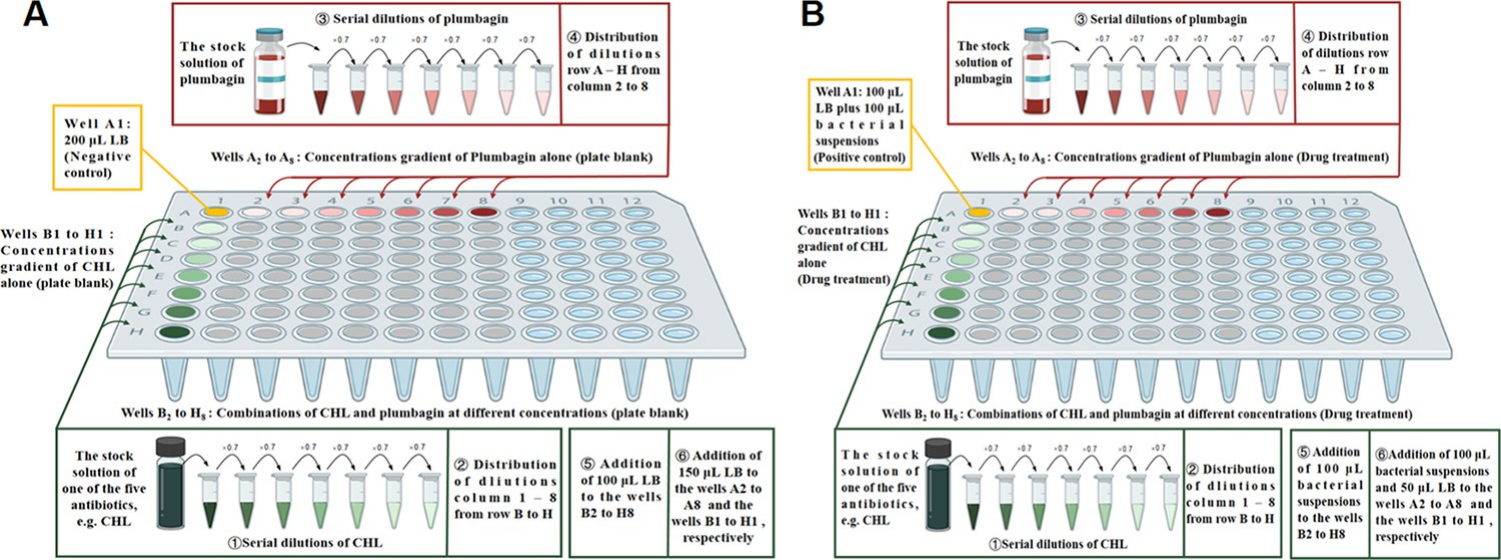
Example of microplate configurations for two-drug dilution assays. (**A**) The experimental plate (taking CHL as an example). (**B**) The control plate. CHL: chloramphenicol; PLU: plumbagin.

Two-drug dilution assays were performed in 96-well microtiter plates, which included an experimental plate and a control plate. In each assay, PLU was paired with each of the five common antibiotics. The plate configuration was then described according to Fig 1. Briefly, for the experimental plate, well A1 served as the positive control and contained 100 μL LB plus 100 μL bacterial suspensions. One of the five antibiotics (50 μL) by the concentration of 0.343×IC_50_ was added to Row B (B1–B8) and followed by 50 μL of each concentration: 0.49×IC_50_, 0.7×IC_50_, 1.0×IC_50_, IC_50_÷0.7, IC_50_÷0.49, and IC_50_÷0.343 of this antibiotic for addition to the next row down, etc. For PLU, the process was then repeated, beginning with Column 2 (A2–H2) and continuing to Column 8. However, the concentrations of PLU were 0.343×MIC, 0.49×MIC, 0.7×MIC, 1.0×MIC, MIC÷0.7, MIC÷0.49, and MIC÷0.343, respectively. The wells of the matrix (B2–H8), loaded with 100 μL bacterial suspensions, were assigned to the crosswise 7×7. The wells of Row A (A2–A8) and Columns 1 (B1–H1), added to 100 μL bacterial suspensions and 50 μL LB, were reserved for dose-response curves of PLU and the antibiotic, respectively.

For the control plate, the dual drug crosswise dilution matrix assays were performed in a similar format as the experimental plates. The only difference was the substitution of 100 μL LB for 100 μL bacterial suspensions. Well A1 contains 200 μL of LB as the negative control. The wells of the matrix (B2–H8), Row A (A2–A8), and Columns 1 (B1–H1) were used in the plate blank.

The plates were incubated with shaking at 37°C, and the bacterial growth was assessed according to Eq (1). Each experiment was conducted at least three times.

### 2.6. Statistical analysis

The calculation and visualization of the plate data were performed by the approach of response surface analysis. This approach is based on the Bliss independence model, and calculations are performed using Combenefit software version 2.021 (Cancer Research UK Cambridge Institute, Cambridge, UK) [26]. The dose-response curve for each of the five common antibiotics and PLU alone was modeled based on the experimental plate data, which was expressed as a percentage of bacterial growth. From these dose-response curves, the reference response surface of an indifferent interaction between two drugs was calculated according to the Bliss independence model. This response surface was then compared to the experimental response surface by Combenefit software, and a graphical representation of the synergy levels can be mapped onto the experimental combination dose-response surface. In addition to the graphical output, three metrics (SYN_SUM, ANT_SUM, and SUM_SYN_ANT) were generated to quantitatively assess these interactions.

## 3. Results

### 3.1. Antibacterial activity of plumbagin and each of the five common antibiotics

**Fig 2:**
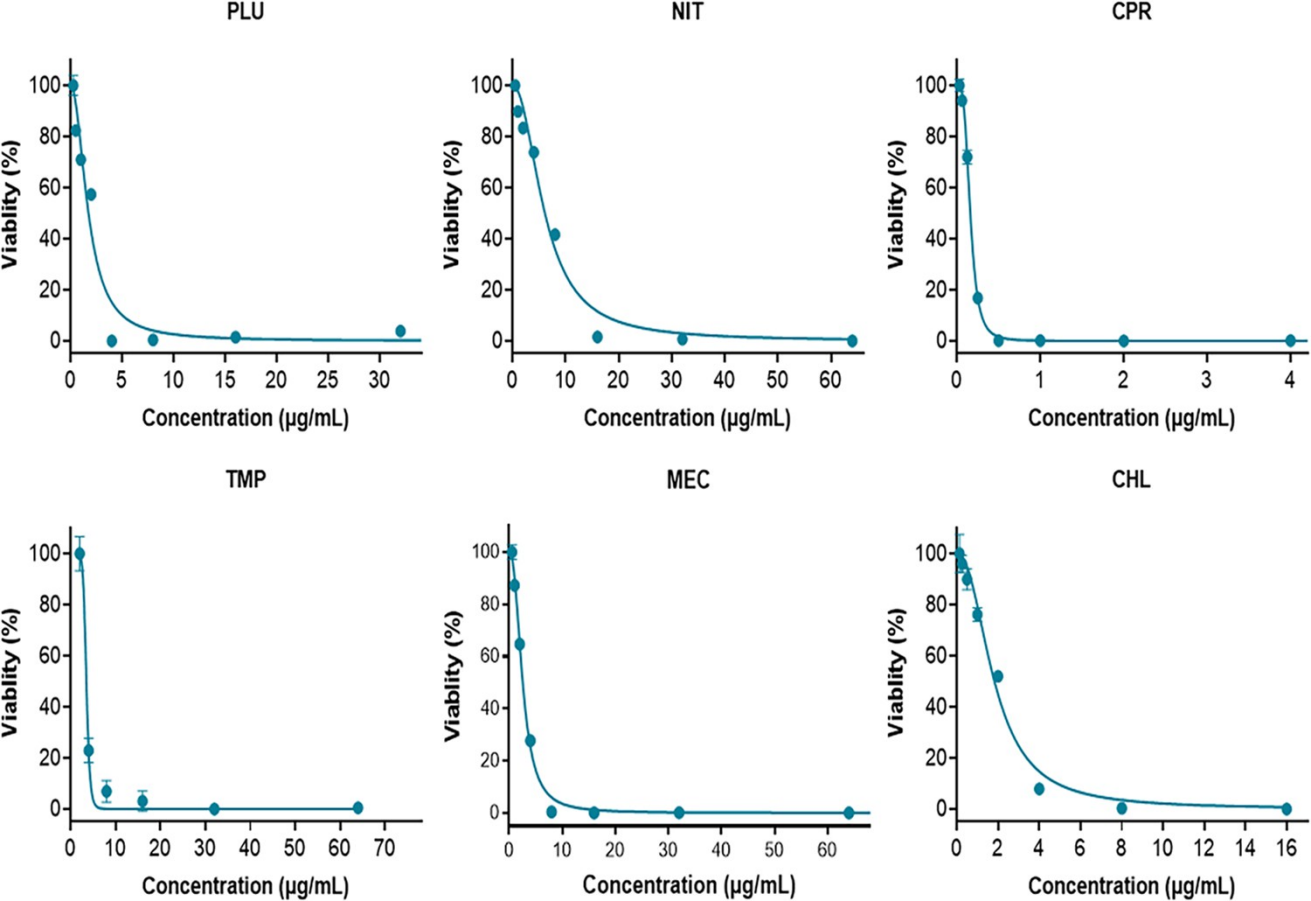
Dose-response curves of PLU and antibiotics against *S*. *aureus in vitro* using GraphPad Prism 9.0. PLU: plumbagin, NIT: nitrofurantoin, CPR: ciprofloxacin, MEC: mecillinam, CHL: chloramphenicol, TMP: trimethoprim.

The dose responses of PLU and each of the five antibiotics are shown in Fig 2. The individual MICs and IC_50_ values of PLU, NIT, CPR, MEC, TMP, and CHL were summarized in Table 1. The active constituent PLU has shown potent antibacterial activity, with median IC_50_ and MIC values against *S*. *aureus* of 1.73 and 4 μg/mL, respectively. The mean IC_50_ and MIC values of the five common antibiotics (NIT, CPR, MEC, TMP, and CHL) against *S*. *aureus* were as follows: 6.10 μg/mL vs. 16 μg/mL, 0.16 μg/mL vs. 0.5 μg/mL, 2.55 μg/mL vs. 8 μg/mL, 3.50 μg/mL vs. 8 μg/mL, and 1.82 μg/mL vs. 4 μg/mL, respectively. Raw data of these experiments can be found in (S1 File).

### 3.2. Calculating of the synergy distribution using three metrics

**Fig 3:**
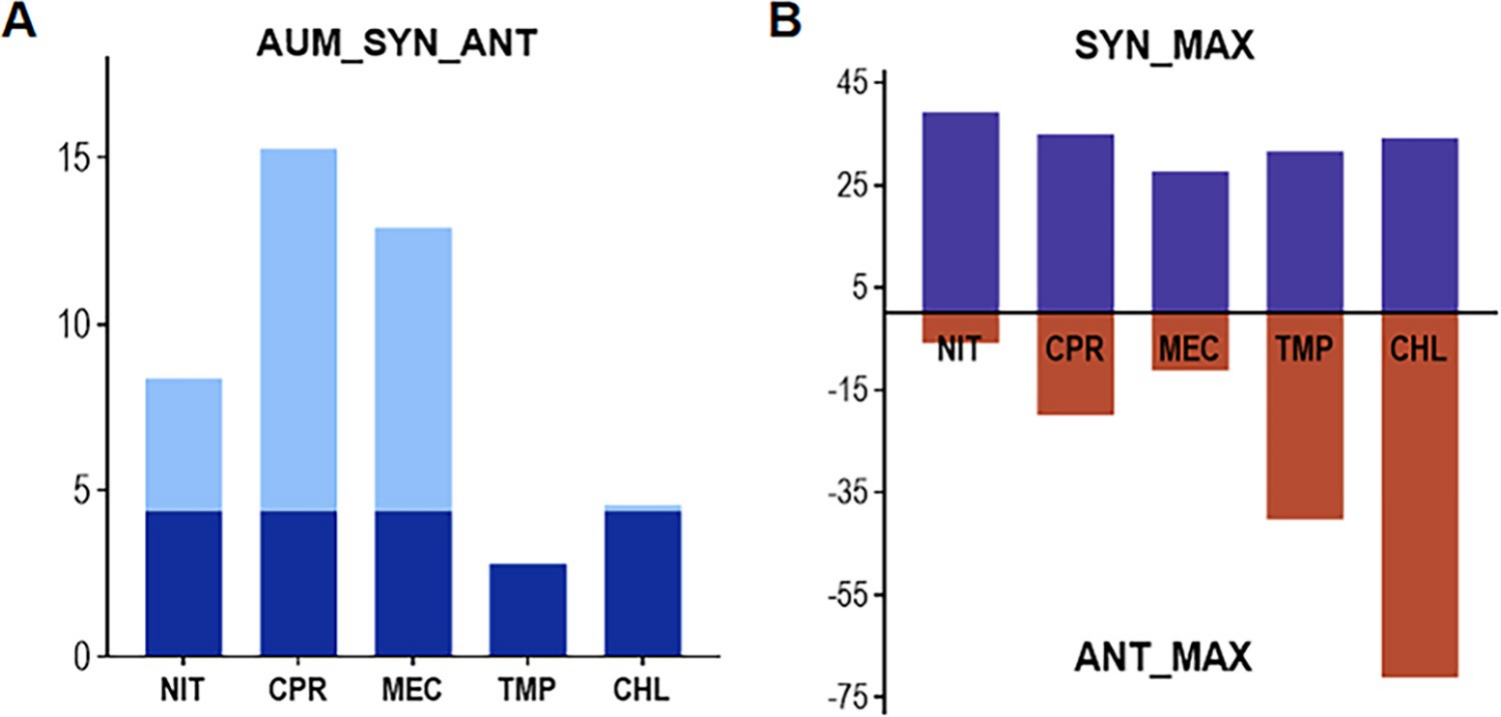
Graphical representation of three metrics generated by the Combenefit software for each of the five antibiotics paired with PLU. (**A**) The SUM_SYN_ANT metric was defined as the “sum of synergy and antagonism observed,” representing all values within the dose space. The light blue section showed that the observed synergy was greater than the combination with PLU itself; (**B**) The SYN_MAX metric is defined as the "maximum observed synergy," which represents the highest value of synergy observed. The ANT_MAX metric was defined as the "maximum antagonism observed," representing the highest value of antagonism recorded. PLU: plumbagin, NIT: nitrofurantoin, CPR: ciprofloxacin, MEC: mecillinam, CHL: chloramphenicol, TMP: trimethoprim.

To assess the effect of drug interactions between a specific pair of drugs, the Combenefit software package calculates the distributions of synergy and antagonism and computes various metrics based on these distributions. The three metrics, SYN_SUM, ANT_SUM, and SUM_SYN_ANT, are shown in Fig 3.

**Fig 4:**
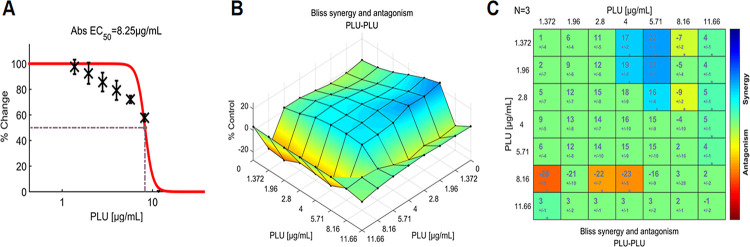
The interactions of PLU with itself (N = 3) are analyzed by the Bliss independence model to determine the threshold of the metric. (**A**) Single-agent dose-response curve for PLU; (**B**) The interaction landscape of the combinations of PLU with itself, visualizing the efficacy of each of the 49 dual-drug combinations. The plot was colored without regard to statistical significance, according to the Synergism/Antagonism scale located just below the graph; (**C**) Synergy scores, calculated by the Bliss independence model, were shown in matrix format. The larger numeral in each box was the synergy score. The number below the synergy score was the standard deviation. The boxes, colored according to the synergism/antagonism scale, indicate results that were statistically significant based on the one-sample t-test. The degrees of significance were as follows: *p<0.05; **p<0.001. If the synergy score is not significant, the box is shown in green; significant is shown in blue; and significant antagonism is shown in red. The number of biological replicates (N) is indicated in the top left of the matrix. Raw data of these experiments can be found in (S2 File). PLU: plumbagin.

To determine the threshold of the metric, the interaction of PLU with itself was tested experimentally in triplicate. Based on these results (Fig 4), synergy was concluded when SUM_SYN_ANT was greater than +4.39, and antagonism was assumed when it was less than -4.39. Between -4.39 and +4.39, indifference was considered.

Based on the SUM_SYN_ANT metric shown in Fig 3A and the threshold described above, NIT (+8.38), CPR (+15.31), MEC (+12.90), and CHL (+4.55) each achieved a net synergistic effect when combined with PLU. TMP (+2.79), with a range of -4.39 to +4.39, was considered to be within the range of indifference. None of the five common antibiotics, with scores below -4.39, exhibited strong antagonism when combined with PLU.

**Fig 5:**
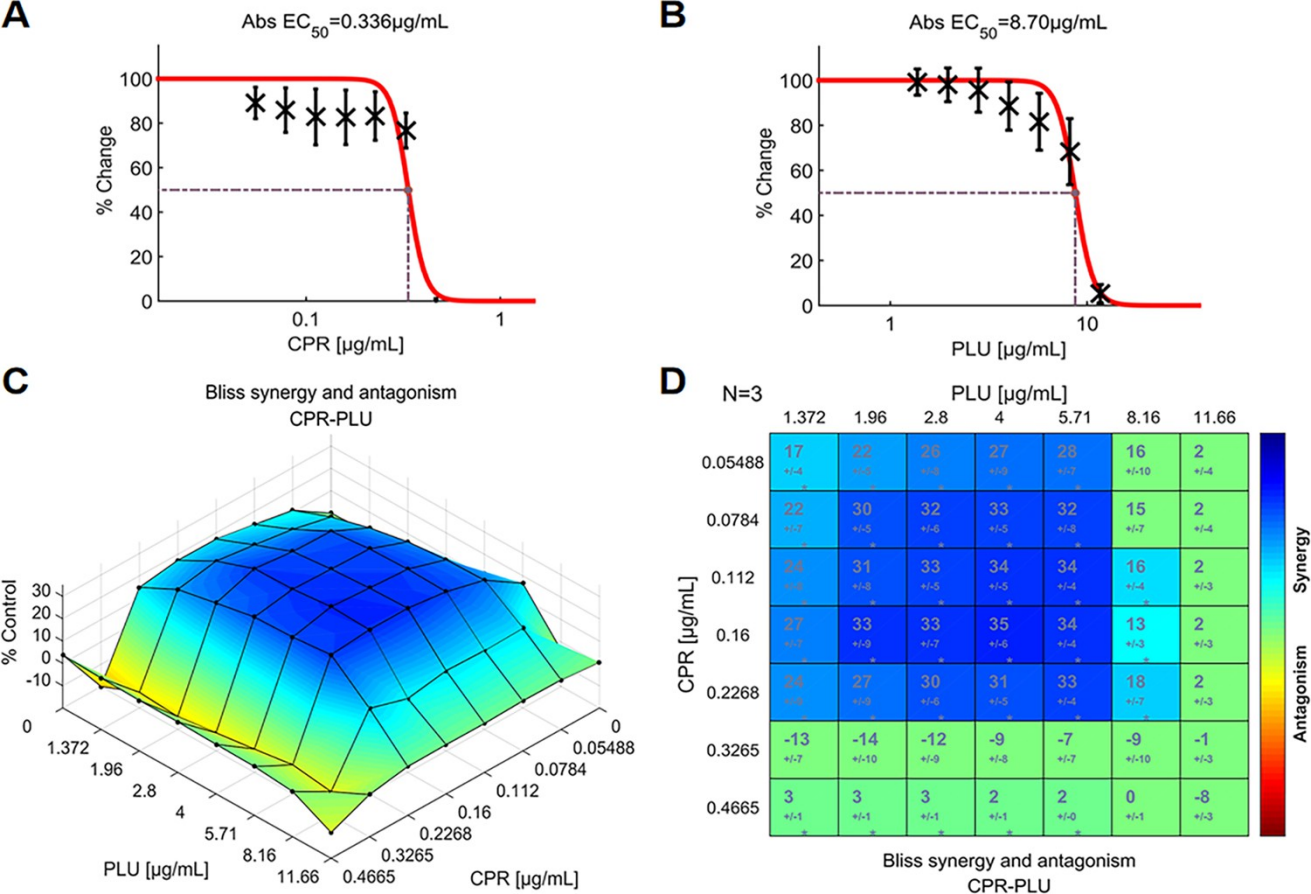
The interactions of CPR and PLU (N = 3), indicate evident synergy between the two agents. (**A**) Single-agent dose-response curve for CPR; (**B**) Single-agent dose-response curve for PLU. The Bliss independence model generated the reference concentration space from the two single-agent dose-response curves; (**C**) The interaction landscape of the combinations of CPR and PLU; (**D**) Synergy scores were calculated by the Bliss independence model and were colored when they are significant. A detailed description of the various plots is given in Fig 4. Raw data of these experiments can be found in (S2 File). CPR: ciprofloxacin, PLU: plumbagin.

CPR, in combination with PLU, had the second-highest SYN_MAX score of +35.00 (Figs 3B and 5D). However, it achieved the highest composite score of +15.31 (Fig 3A). The synergism was evident across a wide range of concentrations of CPR (0.05488–0.2268 μg/mL) and PLU (1.372–5.71 μg/mL). A score of +35 (P<0.05) has resulted in a concentration of 0.16 μg/mL CPR paired with 4 μg/mL PLU (Fig 5D). From the dose-response surface (Fig 5C), it is evident that the combination of CPR and PLU resulted in a significant inhibition of growth, demonstrating a synergistic effect. It was apparent from this plot that there were no significant antagonistic interactions.

**Fig 6:**
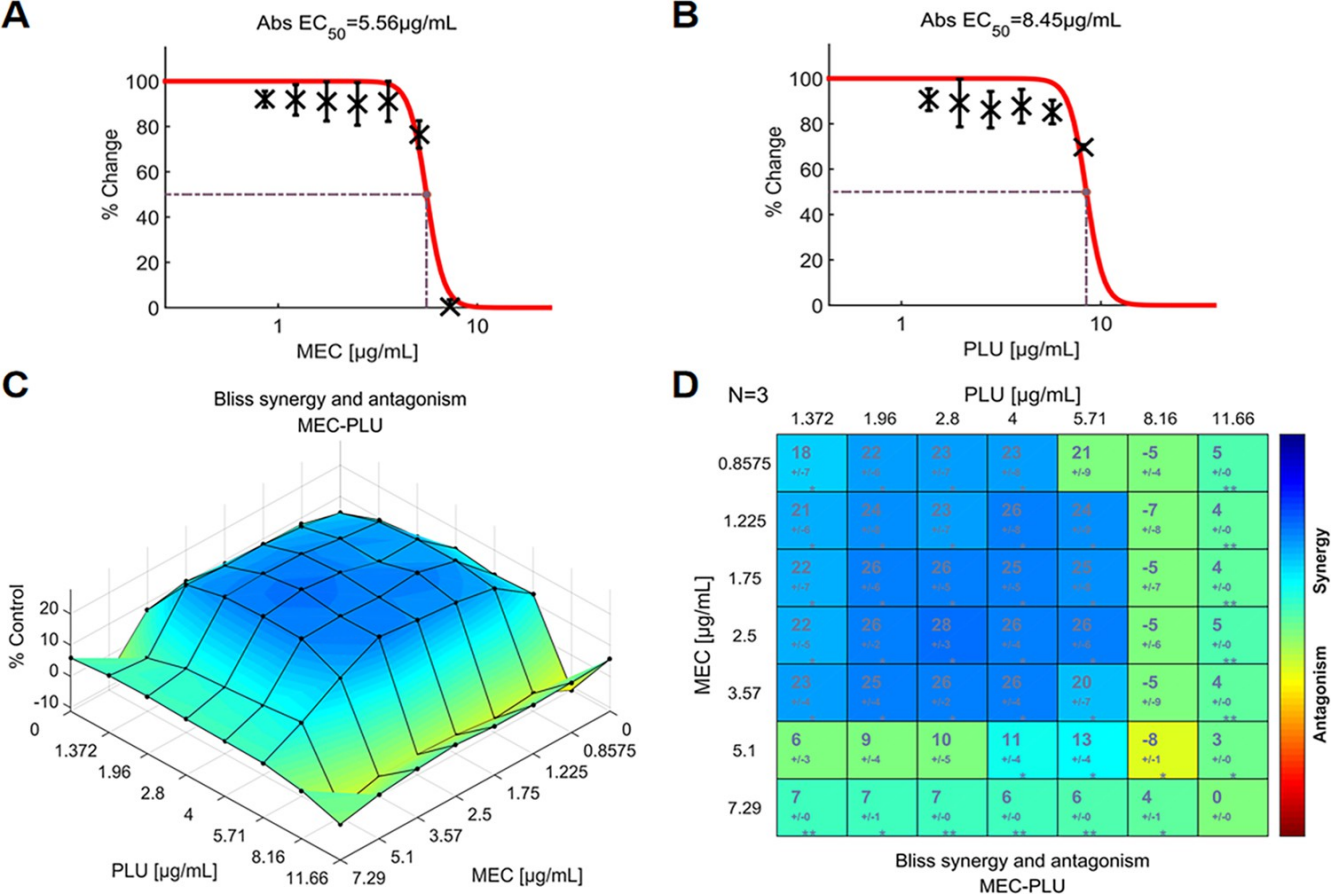
The interactions of MEC and PLU (N = 3), indicate evident synergy between the two agents. (**A**) Single-agent dose-response curve for MEC; (**B**) Single-agent dose-response curve for PLU; (**C**) The interaction landscape of the combinations of MEC and PLU; (**D**) Synergy scores calculated by the Bliss independence model and colored when significant. A detailed description of the various plots is given in Fig 4. Raw data of these experiments can be found in (S2 File). MEC: mecillinam, PLU: plumbagin.

The combination of MEC with PLU achieved the second-highest composite score of +12.90 (Fig 3A). The maximum score for synergism was +28 with the 2.5 μg/mL MEC and 2.8 μg/mL PLU (Fig 6D). Just like in the case of CPR, a significant synergistic effect of MEC was found in a wide range of concentrations (0.8575–3.57 μg/mL) when combined with PLU (Fig 6D). The combination dose-response surface is shown in Fig 6C. From this plot, the synergistic matrix for MEC exhibited a similar pattern to the CPR plus PLU.

**Fig 7:**
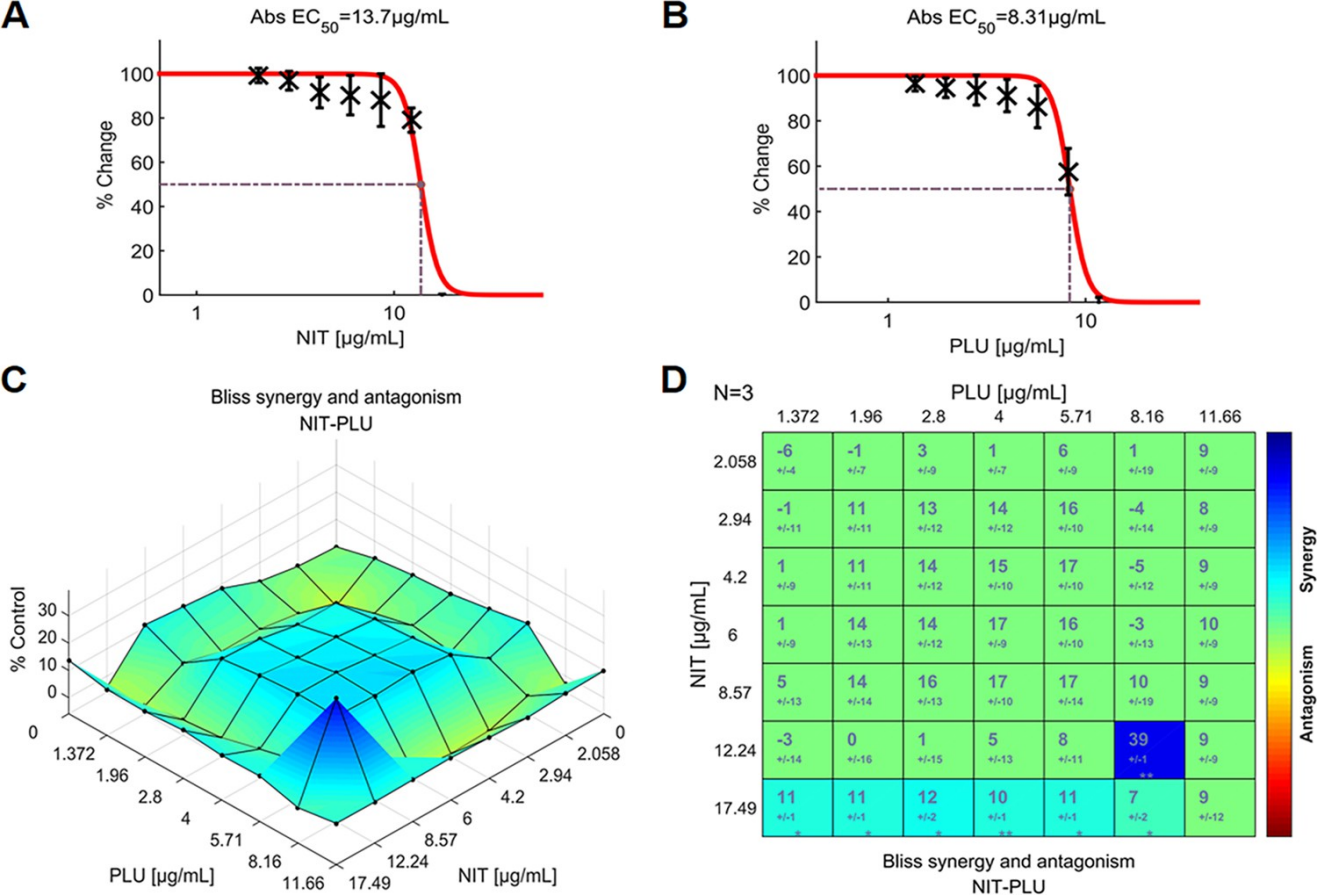
The interactions of NIT and PLU (N = 3), indicate modest synergy between the two agents. (**A**) Single-agent dose-response curve for NIT; (**B**) Single-agent dose-response curve for PLU; (**C**) The interaction landscape of the combinations of NIT and PLU; (**D**) Synergy scores were calculated by the Bliss independence model and were colored when significant. A detailed description of the various plots is given in Fig 4. Raw data of these experiments can be found in (S2 File). NIT: nitrofurantoin, PLU: plumbagin.

Although the NIT-PLU pairing had the highest SYN_MAX score of +39.23 (Figs 3B and 7D), the interactions between NIT and PLU were statistically insignificant over more than 90% of the concentration range. Moreover, this combination exhibited a modest SUM_SYN_ANT score of +8.38 (Fig 3A). The score of +39 (P<0.01), obtained from the interaction between 12.24 μg/mL of NIT and 8.16 μg/mL of PLU (Fig 7D), made a significant contribution to this result. Furthermore, another reason was that there was no significant antagonistic interaction, as shown in Fig 7.

**Fig 8:**
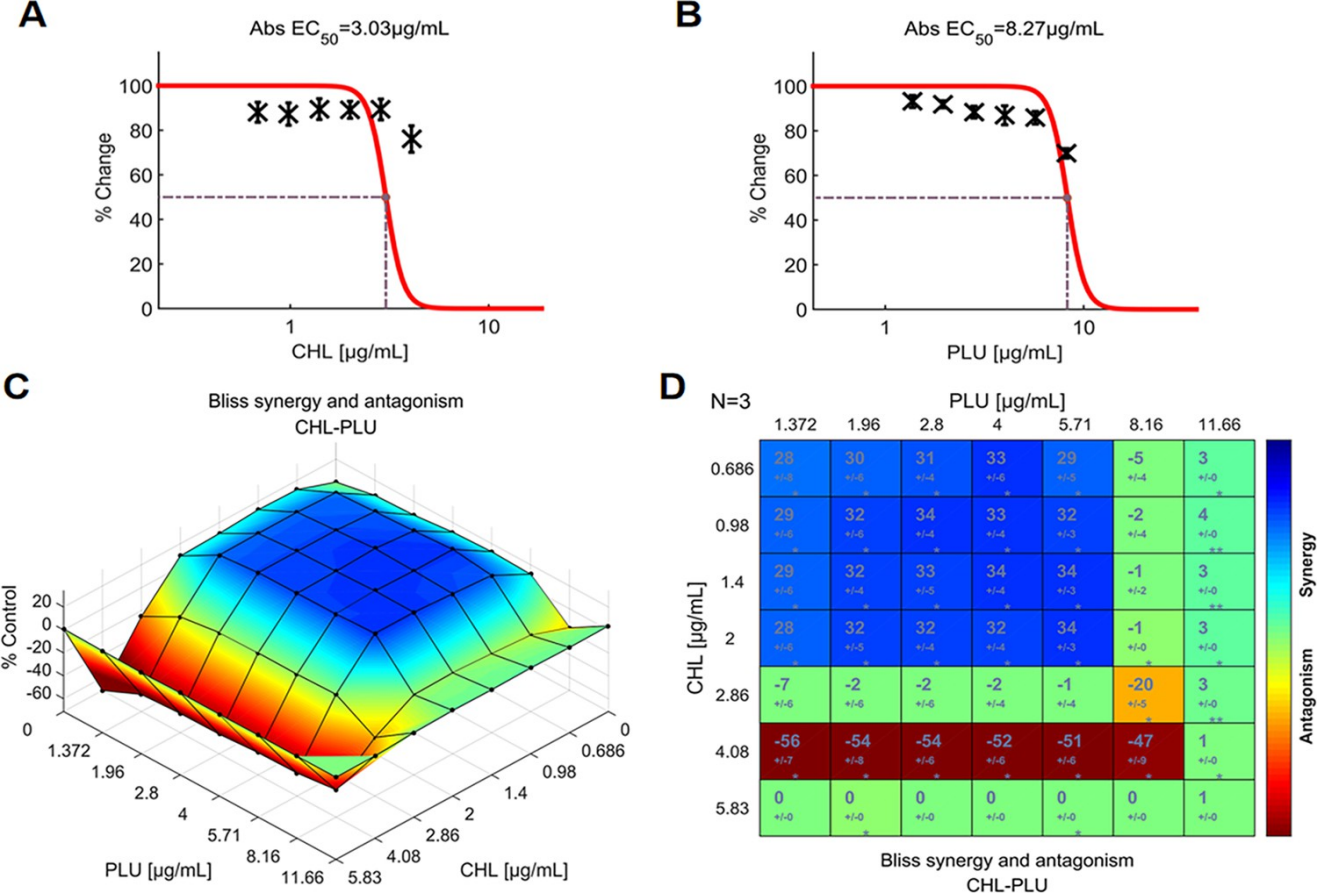
The interactions of CHL and PLU (N = 3), indicate indifference between the two agents. (**A**) Single-agent dose-response curve for CHL; (**B**) Single-agent dose-response curve for PLU; (**C**) The interaction landscape of the combinations of CHL and PLU; (**D**) Synergy scores were calculated by the Bliss independence model and were colored when significant. A detailed description of the various plots is given in Fig 4. Raw data of these experiments can be found in (S2 File). CHL: chloramphenicol, PLU: plumbagin.

The SUM_SYN_ANT score for the CHL-PLU pairing was +4.55, and the maximum scores for synergism and antagonism were +34.18 and -71.19, respectively (Fig 3). The pattern of CHL-PLU interaction revealed a coexistence of stronger antagonism and synergism across the entire concentration range (Fig 8C and 8D). Importantly, the lower concentrations of CHL (0.686–2 μg/mL) synergistically interacted with PLU (1.372–5.71 μg/mL), while the higher concentrations of CHL antagonistically interacted. The 4.08 μg/mL CHL demonstrated statistically significant antagonism across almost the entire concentration range of PLU. Thus, it can be observed that the significant antagonism and synergism largely offset each other in the calculation of the SUM_SYN_ANT score. These observations were reinforced by the experimental dose-response space plots.

### 3.3. Evaluation of the indifference or antagonism between antibiotics and plumbagin

**Fig 9:**
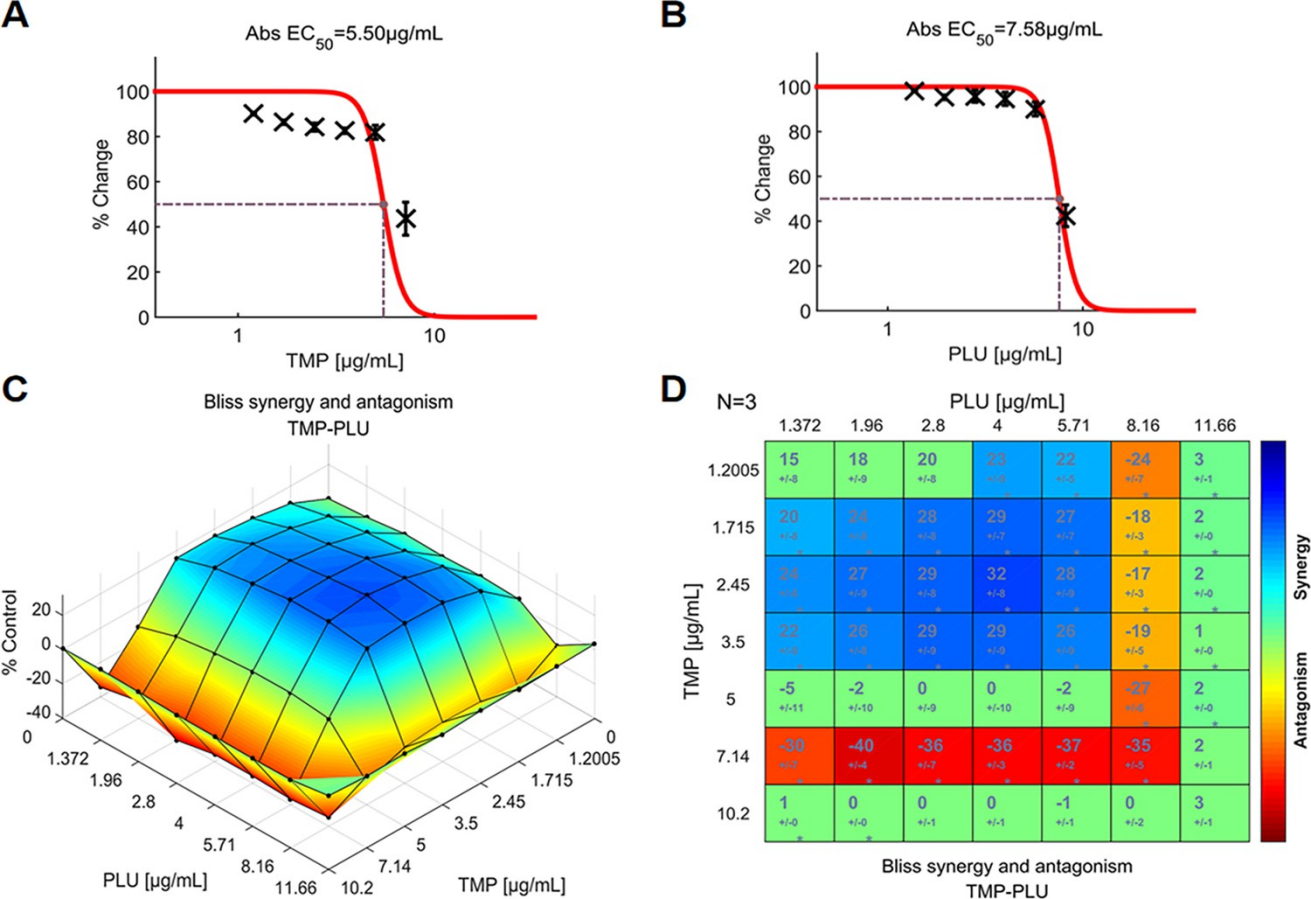
The interactions of TMP and PLU (N = 3), indicate indifference between the two agents. (**A**) Single-agent dose-response curve for TMP; (**B**) Single-agent dose-response curve for PLU; (**C**) The interaction landscape of the combinations of TMP and PLU; (**D**) Synergy scores were calculated by the Bliss independence model and were colored when significant. A detailed description of the various plots is given in Fig 4. Raw data of these experiments can be found in (S2 File). TMP: trimethoprim, PLU: plumbagin.

None of the five drug combinations with scores below -4.39 demonstrated strong antagonism within the concentration matrix (Fig 3A). TMP, in combination with PLU, achieved the lowest composite score of +2.79 (Fig 3A), although it also had a higher SYN_MAX score of +31.67 (Fig 3B). Moreover, the synergism was evident across a wide range of concentrations (1.372–5.71 μg/mL) and TMP concentrations (1.2–3.5 μg/mL). The dose-response surface is shown in Fig 9D. From this plot, it can be observed that a higher concentration of TMP, when combined with almost all concentrations of PLU, resulted in a significant antagonistic effect. Similarly, higher concentrations of PLU showed significant antagonism with almost all concentrations of TMP. These antagonistic effects largely offset the significant synergies between TMP and PLU.

## 4. Discussion

During the development of new antibiotics, the search for effective drug combinations has been recognized as an attractive strategy to reduce the opportunity for bacteria to develop resistance and achieve a successful treatment outcome. To our knowledge, we have performed the initial analysis of PLU paired with each of five common antibiotics, which have different mechanisms and varying levels of activity against *S*. *aureus* (Table 1). Synergistic interactions were observed against *S*. *aureus in vitro* when PLU was combined with NIT, CPR, MEC, and CHL. A single PLU exhibited antimicrobial activity against *S*. *aureus*, and the observed MIC in this study (4 μg/mL) was consistent with previously published data. The Paiva SR [27] study showed that the MIC of PLU was 1.56 μg/mL, while Rondevaldova [17] showed that the MIC of PLU was 8 μg/mL. Additionally, it was found that the combination of PLU with oxacillin and tetracycline had a synergistic effect. These findings are encouraging since PLU not only has great potential in the development of antibacterial drugs, but it is also a promising compound with the potential to work in combination with antibiotics that have different mechanisms. Chen [28] observed a synergistic effect of the combination of PLU and gentamicin in treating *Klebsiella pneumoniae*. The group that received the combined treatment showed higher intracellular gentamicin concentration, increased membrane potential, and enhanced proton motility. Further metabolomics analysis indicated that the combination may also achieve synergistic antibacterial effects by enhancing the tricarboxylic acid cycle. However, the mechanism is the combination of PLU and antibiotics, rather than the mechanism of PLU alone [28, 29]. While PLU does possess antibacterial activity, further investigation is needed to determine its specific mechanism of action against bacteria.

PLU possesses high biological activity, as described in several previous studies [30–33]. The mode of action for the antibacterial activity of PLU may involve the enhancement of reactive oxygen species (ROS) [34]. The generation of reactive oxygen species (ROS) leads to the inhibition of cellular enzymes involved in apoptotic cell death. Furthermore, the carbonyl groups at C1 and C4 of PLU enhance its antimicrobial activity. The core of the two rings is essential for antimicrobial activity. Because of its structural characteristics, PLU easily interacts with nucleophilic amino acids found in proteins. This interaction often leads to the inactivation of proteins and the subsequent loss of their function [35]. On the other hand, in the case of *S*. *aureus*, the likely targets are surface-exposed adhesins, cell wall polypeptides, and membrane-bound enzymes [36]. Given these facts, the performance of the antimicrobial activity of PLU and its combination with antibiotics might be attributed to its role as a non-selective inhibitor of certain proteins.

This study is intended to evaluate the efficacy of PLU paired with each of the five common antibiotics against *S*. *aureus*, an important consideration in the experimental design was the selection of appropriate statistical models. Currently, the evaluation of drug combination efficacy and synergy classification involves the use of the Loewe additivity and Bliss independence models [37, 38]. These two models have been discussed by Baeder [39] and Vakil [40]. However, there is still no standardized guideline on how to choose the optimal model [41]. In practice, when the dose-response curves of individual drugs are well characterized, the Loewe additivity model works well. However, when the dose-response curves are nonstandard, the reliability of the Loewe additivity model may be compromised. In such cases, the Bliss independence model can provide a viable alternative. Further, the Bliss independence model assumes a stochastic process in which two drugs elicit their effects independently [24]. Due to the different modes of action between PLU and each of the five common antibiotics, including interactions with different targets or signaling pathways, we assumed that the actions of PLU and antibiotics against *S*. *aureus* were independent. Therefore, we chose the Bliss independence model as more appropriate.

According to the SUM_SYN_ANT metric of the Combenefit program, four out of five antibiotics (NIT, CPR, MEC, and CHL) paired with PLU showed significant synergy. Only one antibiotic (TMP) had an indifferent interaction with PLU. The metrics may be sufficient for initially filtering potential synergistic drug combinations. However, as previously described, different concentration ratios can result in varying interactions within the same drug combination [42]. Excessive dependence on the SUM_SYN_ANT metrics may potentially overlook synergistic interactions that could be beneficial, especially when these interactions are confined to specific dose regions and frequently accompanied by antagonism (Figs 8D and 9D). A comparison of the interactions between NIT-PLU and CHL-PLU pairings further illustrates the flaw of metrics. The SUM_SYN_ANT metric for NIT (+8.38) is higher than for CHL (+4.55). However, the combination of CHL and PLU could occur as one of the most significant synergistic interactions within specific dosage ranges. Moreover, such synergistic interactions found at lower concentrations of both drugs may have greater clinical value.

In addition, a follow-up confirmatory screen usually uses dose-response matrices [43], which are more informative, especially when synergism and antagonism occur in the same dose region. In the dose-response matrix, the Bliss synergy score can be calculated for each dose combination, allowing a surface plot. The interaction landscape of drug combinations can be visualized using surface plots, with the aim of identifying synergistic and antagonistic dosage regions for further dose optimization in a validation screen [44].

For the CPR and PLU combination, the interaction landscapes were shown in both 3D and 2D (Fig 5C and 5D). Such a landscape pattern indicates that the dosage of CPR can be reduced threefold while maintaining the same level of response. On the other hand, the analysis of the interaction landscape also revealed a distinct antagonistic effect within a specific dose region. As can be seen from Fig 8D, the antagonism is centered around the combination of CHL at a dose of 4.08 μg/mL. Antagonistic effects are often overlooked in treatment, but they can promote maximum efficacy by maintaining synergies and avoiding antagonistic ratios when combining drugs [45]. This provides clinically meaningful guidelines for the application of drug combinations. Furthermore, studying these combinations may provide valuable insights into the interconnected mechanisms of *S*. *aureus* signaling pathways [46–48].

## 5. Conclusion

Overall, this study demonstrated that PLU is not only a potent anti-*S*. *aureus* compound, but it can also enhance the growth-inhibitory effects of NIT, CPR, MEC, and CHL against *S*. *aureus*. Although the effect of TMP-PLU pairing was indifferent across the entire dose-response matrix, we observed the highest synergy within a specific dose region based on the analysis of the interaction landscape. In addition, no antagonistic interactions were indicated. Based on these results, the PLU paired with each of the five common antibiotics, which have various mechanisms of action, could be considered as an agent that produces a synergistic anti-*S*. *aureus* effect within a specific dosage range.

Although standardization has to be improved, the method used in this study, which is based on the Bliss independence model and implemented with the Combenefit software package, is robust. The use of the interaction landscape, along with the full dose-response matrix and the SUM_SYN_ANT metrics, has proven to be an effective method for analyzing and quantifying combination results in antibacterial research. This approach provides a better understanding of antibacterial combinations. Therefore, significant results can be obtained from this study. Further research focused on understanding the mechanisms of action in combination therapy for clinical translation will be necessary. This includes using multiple techniques and forms of analysis to interpret the results before practical application can be considered.

## Supporting information

S1 File Bacterial survival data of MICs and IC50s tests. (XLSX)

S2 File Bacterial survival data in drug interactions. (XLSX)

## References

[1] Cheung GYC, Bae JS, Otto M. Pathogenicity and virulence of *Staphylococcus aureus*. Virulence. 2021;12: 547–569. doi: 10.1080/21505594.2021.1878688 33522395 PMC7872022

[2] Dayan GH, Mohamed N, Scully IL, Cooper D, Begier E, Eiden J, et al. *Staphylococcus aureus*: the current state of disease, pathophysiology and strategies for prevention. Expert Rev Vaccines. 2016;15: 1373–1392. doi: 10.1080/14760584.2016.1179583 27118628

[3] Foster TJ. Antibiotic resistance in *Staphylococcus aureus*. Current status and future prospects. FEMS Microbiology Reviews. 2017;41: 430–449. doi: 10.1093/femsre/fux007 28419231

[4] Guo Y, Song G, Sun M, Wang J, Wang Y. Prevalence and Therapies of Antibiotic-Resistance in *Staphylococcus aureus*. Front Cell Infect Microbiol. 2020;10: 107. doi: 10.3389/fcimb.2020.00107 32257966 PMC7089872

[5] Mlynarczyk-Bonikowska B, Kowalewski C, Krolak-Ulinska A, Marusza W. Molecular Mechanisms of Drug Resistance in *Staphylococcus aureus*. Int J Mol Sci. 2022;23: 8088. doi: 10.3390/ijms23158088 35897667 PMC9332259

[6] Bhattacharya R, Rolta R, Dev K, Sourirajan A. Synergistic potential of essential oils with antibiotics to combat fungal pathogens: Present status and future perspectives. Phytotherapy Research. 2021;35: 6089–6100. doi: 10.1002/ptr.7218 34324240

[7] Bremner JB. An Update Review of Approaches to Multiple Action-Based Antibacterials. Antibiotics. 2023;12: 865. doi: 10.3390/antibiotics12050865 37237768 PMC10215433

[8] Pennings LJ, Ruth MM, Wertheim HFL, van Ingen J. The Benzimidazole SPR719 Shows Promising Concentration-Dependent Activity and Synergy against Nontuberculous Mycobacteria. Antimicrob Agents Chemother. 2021;65: e02469–20. doi: 10.1128/AAC.02469-20 33468478 PMC8097464

[9] Bittner Fialová S, Rendeková K, Mučaji P, Nagy M, Slobodníková L. Antibacterial Activity of Medicinal Plants and Their Constituents in the Context of Skin and Wound Infections, Considering European Legislation and Folk Medicine—A Review. IJMS. 2021;22: 10746. doi: 10.3390/ijms221910746 34639087 PMC8509446

[10] Nafis A, Kasrati A, Jamali CA, Custódio L, Vitalini S, Iriti M, et al. A Comparative Study of the in Vitro Antimicrobial and Synergistic Effect of Essential Oils from Laurus nobilis L. and Prunus armeniaca L. from Morocco with Antimicrobial Drugs: New Approach for Health Promoting Products. Antibiotics (Basel). 2020;9: 140. doi: 10.3390/antibiotics9040140 32218155 PMC7235724

[11] Puljula E, Walton G, Woodward MJ, Karonen M. Antimicrobial Activities of Ellagitannins against *Clostridiales perfringens*, *Escherichia coli*, *Lactobacillus plantarum* and *Staphylococcus aureus*. Molecules. 2020;25: 3714. doi: 10.3390/molecules25163714 32824081 PMC7465317

[12] Reddy PJ, Ray S, Sathe GJ, Prasad TSK, Rapole S, Panda D, et al. Proteomics Analyses of *Bacillus subtilis* after Treatment with Plumbagin, a Plant-Derived Naphthoquinone. OMICS: A Journal of Integrative Biology. 2015;19: 12–23. doi: 10.1089/omi.2014.0099 25562197 PMC4281856

[13] Rajakrishnan R, Lekshmi R, Benil PB, Thomas J, AlFarhan AH, Rakesh V, et al. Phytochemical evaluation of roots of Plumbago zeylanica L. and assessment of its potential as a nephroprotective agent. Saudi J Biol Sci. 2017;24: 760–766. doi: 10.1016/j.sjbs.2017.01.001 28490944 PMC5415120

[14] Tripathi SK, Panda M, Biswal BK. Emerging role of plumbagin: Cytotoxic potential and pharmaceutical relevance towards cancer therapy. Food and Chemical Toxicology. 2019;125: 566–582. doi: 10.1016/j.fct.2019.01.018 30685472

[15] Shao Y, Dang M, Lin Y, Xue F. Evaluation of wound healing activity of plumbagin in diabetic rats. Life Sciences. 2019;231: 116422. doi: 10.1016/j.lfs.2019.04.048 31059689

[16] Janeczko M, Demchuk OM, Strzelecka D, Kubiński K, Masłyk M. New family of antimicrobial agents derived from 1,4-naphthoquinone. European Journal of Medicinal Chemistry. 2016;124: 1019–1025. doi: 10.1016/j.ejmech.2016.10.034 27783973

[17] Rondevaldova J, Novy P, Kokoska L. In vitro combinatory antimicrobial effect of plumbagin with oxacillin and tetracycline against *Staphylococcus aureus*. Phytother Res. 2015;29: 144–147. doi: 10.1002/ptr.5237 25266704

[18] Yap JKY, Tan SYY, Tang SQ, Thien VK, Chan EWL. Synergistic Antibacterial Activity Between 1,4-Naphthoquinone and β-Lactam Antibiotics Against Methicillin-Resistant *Staphylococcus aureus*. Microb Drug Resist. 2021;27: 234–240. doi: 10.1089/mdr.2020.0178 32589487

[19] Hall MJ, Middleton RF, Westmacott D. The fractional inhibitory concentration (FIC) index as a measure of synergy. J Antimicrob Chemother. 1983;11: 427–433. doi: 10.1093/jac/11.5.427 6874629

[20] Yadav B, Wennerberg K, Aittokallio T, Tang J. Searching for Drug Synergy in Complex Dose–Response Landscapes Using an Interaction Potency Model. Computational and Structural Biotechnology Journal. 2015;13: 504–513. doi: 10.1016/j.csbj.2015.09.001 26949479 PMC4759128

[21] Chou T-C, Talalay P. Quantitative analysis of dose-effect relationships: the combined effects of multiple drugs or enzyme inhibitors. Advances in Enzyme Regulation. 1984;22: 27–55. doi: 10.1016/0065-2571(84)90007-4 6382953

[22] Chou T-C. Theoretical Basis, Experimental Design, and Computerized Simulation of Synergism and Antagonism in Drug Combination Studies. Pharmacol Rev. 2006;58: 621–681. doi: 10.1124/pr.58.3.10 16968952

[23] Chou T-C. Drug Combination Studies and Their Synergy Quantification Using the Chou-Talalay Method. Cancer Research. 2010;70: 440–446. doi: 10.1158/0008-5472.CAN-09-1947 20068163

[24] Bliss CI. THE TOXICITY OF POISONS APPLIED JOINTLY1. Annals of Applied Biology. 1939;26: 585–615. doi: 10.1111/j.1744-7348.1939.tb06990.x

[25] Ma J, Motsinger-Reif A. Current Methods for Quantifying Drug Synergism. Proteom Bioinform. 2019;1: 43–48. doi: 10.2203/dose-response.09-030.Beam 32043089 PMC7010330

[26] Di Veroli GY, Fornari C, Wang D, Mollard S, Bramhall JL, Richards FM, et al. Combenefit: an interactive platform for the analysis and visualization of drug combinations. Bioinformatics. 2016;32: 2866–2868. doi: 10.1093/bioinformatics/btw230 27153664 PMC5018366

[27] de Paiva SR, Figueiredo MR, Aragão TV, Kaplan MAC. Antimicrobial activity in vitro of plumbagin isolated from Plumbago species. Mem Inst Oswaldo Cruz. 2003;98: 959–961. doi: 10.1590/s0074-02762003000700017 14762525

[28] Chen X, Yin L, Peng L, Liang Y, Lv H, Ma T. Synergistic Effect and Mechanism of Plumbagin with Gentamicin Against Carbapenem-Resistant *Klebsiella pneumoniae*. Infect Drug Resist. 2020;13: 2751–2759. doi: 10.2147/IDR.S265753 PMC7432958 32884304

[29] Wang Y, Kong J, Zhang X, Liu Y, Huang Z, Yuan L, et al. Plumbagin resurrect colistin susceptible against colistin-resistant *Pseudomonas aeruginosa* in vitro and in vivo. Front Microbiol. 2022;13: 1020652. doi: 10.3389/fmicb.2022.1020652 36274701 PMC9579824

[30] Cao Y-Y, Yu J, Liu T-T, Yang K-X, Yang L-Y, Chen Q, et al. Plumbagin inhibits the proliferation and survival of esophageal cancer cells by blocking STAT3-PLK1-AKT signaling. Cell Death Dis. 2018;9: 17. doi: 10.1038/s41419-017-0068-6 29339720 PMC5833725

[31] Kuan-hong W, Bai-zhou L. Plumbagin protects against hydrogen peroxide-induced neurotoxicity by modulating NF-κB and Nrf-2. aoms. 2018;14: 1112–1118. doi: 10.5114/aoms.2016.64768 30154895 PMC6111359

[32] Kapur A, Beres T, Rathi K, Nayak AP, Czarnecki A, Felder M, et al. Oxidative stress via inhibition of the mitochondrial electron transport and Nrf-2-mediated anti-oxidative response regulate the cytotoxic activity of plumbagin. Sci Rep. 2018;8: 1073. doi: 10.1038/s41598-018-19261-w 29348410 PMC5773707

[33] Gupta AC, Mohanty S, Saxena A, Maurya AK, Bawankule DU. Plumbagin, a vitamin K3 analogue ameliorate malaria pathogenesis by inhibiting oxidative stress and inflammation. Inflammopharmacol. 2018;26: 983–991. doi: 10.1007/s10787-018-0465-1 29569058

[34] Mosel M, Li L, Drlica K, Zhao X. Superoxide-Mediated Protection of *Escherichia coli* from Antimicrobials. Antimicrob Agents Chemother. 2013;57: 5755–5759. doi: 10.1128/AAC.00754-13 23979754 PMC3811261

[35] Kumar V, Sharma A, Pratap S, Kumar P. Biochemical and biophysical characterization of 1,4-naphthoquinone as a dual inhibitor of two key enzymes of type II fatty acid biosynthesis from Moraxella catarrhalis. Biochimica et Biophysica Acta (BBA)—Proteins and Proteomics. 2018;1866: 1131–1142. doi: 10.1016/j.bbapap.2018.08.008 30282611

[36] Lucas AL, Manna AC. Phenotypic characterization of sarR mutant in *Staphylococcus aureus*. Microbial Pathogenesis. 2013;57: 52–61. doi: 10.1016/j.micpath.2012.11.008 23183271

[37] Azasi Y, Gallagher SK, Diouf A, Dabbs RA, Jin J, Mian SY, et al. Bliss’ and Loewe’s additive and synergistic effects in *Plasmodium falciparum* growth inhibition by AMA1-RON2L, RH5, RIPR and CyRPA antibody combinations. Sci Rep. 2020;10: 11802. doi: 10.1038/s41598-020-67877-8 32678144 PMC7366652

[38] Wild M, Kicuntod J, Seyler L, Wangen C, Bertzbach LD, Conradie AM, et al. Combinatorial Drug Treatments Reveal Promising Anticytomegaloviral Profiles for Clinically Relevant Pharmaceutical Kinase Inhibitors (PKIs). IJMS. 2021;22: 575. doi: 10.3390/ijms22020575 33430060 PMC7826512

[39] Baeder DY, Yu G, Hozé N, Rolff J, Regoes RR. Antimicrobial combinations: Bliss independence and Loewe additivity derived from mechanistic multi-hit models. Phil Trans R Soc B. 2016;371: 20150294. doi: 10.1098/rstb.2015.0294 27160596 PMC4874391

[40] Vakil V, Trappe W. Drug Combinations: Mathematical Modeling and Networking Methods. Pharmaceutics. 2019;11: 208. doi: 10.3390/pharmaceutics11050208 31052580 PMC6571786

[41] Vlot AHC, Aniceto N, Menden MP, Ulrich-Merzenich G, Bender A. Applying synergy metrics to combination screening data: agreements, disagreements and pitfalls. Drug Discovery Today. 2019;24: 2286–2298. doi: 10.1016/j.drudis.2019.09.002 31518641

[42] Wang P, Zhao L, Huang Y, Qian W, Zhu X, Wang Z, et al. Combined toxicity of nano-TiO2 and Cd2+ to Scenedesmus obliquus: Effects at different concentration ratios. J Hazard Mater. 2021;418: 126354. doi: 10.1016/j.jhazmat.2021.126354 34130160

[43] Park Y, Liu S. On the coherence of model-based dose-finding designs for drug combination trials. PLoS One. 2020;15: e0242561. doi: 10.1371/journal.pone.0242561 33253260 PMC7703981

[44] Twarog NR, Martinez NE, Gartrell J, Xie J, Tinkle CL, Shelat AA. Using response surface models to analyze drug combinations. Drug Discovery Today. 2021;26: 2014–2024. doi: 10.1016/j.drudis.2021.06.002 34119666 PMC8410662

[45] Harasym TO, Liboiron BD, Mayer LD. Drug ratio-dependent antagonism: a new category of multidrug resistance and strategies for its circumvention. Methods Mol Biol. 2010;596: 291–323. doi: 10.1007/978-1-60761-416-6_13 19949929

[46] Chait R, Craney A, Kishony R. Antibiotic interactions that select against resistance. Nature. 2007;446: 668–671. doi: 10.1038/nature05685 17410176

[47] Shavit M, Pokrovskaya V, Belakhov V, Baasov T. Covalently linked kanamycin—Ciprofloxacin hybrid antibiotics as a tool to fight bacterial resistance. Bioorg Med Chem. 2017;25: 2917–2925. doi: 10.1016/j.bmc.2017.02.068 28343755

[48] Saputra EC, Huang L, Chen Y, Tucker-Kellogg L. Combination Therapy and the Evolution of Resistance: The Theoretical Merits of Synergism and Antagonism in Cancer. Cancer Research. 2018;78: 2419–2431. doi: 10.1158/0008-5472.CAN-17-1201 29686021

